# Comparison of Two Passive Samplers for Hydrophilic and Lipophilic Phycotoxins and Expansion of Analytical Methods for Quantification Using UPLC-MS/MS

**DOI:** 10.3390/toxins18100452

**Published:** 2026-10-15

**Authors:** Josh A. Garber, Damian Shea, Juliette L. Smith

**Affiliations:** 1 Virginia Institute of Marine Science, William & Mary, Gloucester Point, VA 23062, USA; jagarber@vims.edu; 2 Statera, Raleigh, NC 27606, USA; dshea@statera.org

**Keywords:** UPLC-MS/MS, harmful algal blooms, cyanotoxins, phycotoxins, passive samplers, mass spectrometry

## Abstract

The co-occurrence of phycotoxins produced by harmful algal blooms is becoming more frequent. To combat this problem, monitoring programs that can track and quantify a wide range of hydrophilic and lipophilic phycotoxins are required. A 24 h, bench-scale study was conducted to provide an initial comparison of apparent sorption and extraction recovery for 24 freshwater and marine phycotoxins from two passive samplers under controlled artificial seawater conditions. Modifications were made to previously published ultra-performance liquid chromatography-tandem mass spectrometry (UPLC-MS/MS) methods to capture all phycotoxin analytes of interest in two injections. This analytical method was also evaluated alongside extraction methods for a commonly used passive sampler, a solid phase adsorption toxin tracker (SPATT), and a novel passive sampler, the composite integrated passive sampler (CIPS). SPATT contained a lipophilic resin, Diaion^®^ HP20, whereas the CIPS is a continuous sheet with hydrophilic and lipophilic regions aimed at allowing for adsorption across a wider range of phycotoxins. A single-step extraction procedure was tested for both samplers to determine apparent extraction recoveries for all 24 phycotoxins. The CIPS seemed to produce overall higher apparent extraction recoveries of analytes, but the receiving phase generally sorbed less ng of phycotoxin during the experimental period than SPATTs and matrix effects were present. Across all lipophilic and hydrophilic phycotoxins, SPATT sorbed more than 90% of each phycotoxin from the seawater within 24 h, for all but four phycotoxins. The CIPS, however, sorbed less of each, but still more than 50% in artificial seawater for all but three phycotoxins. When considering extraction efficiency, SPATT may be better suited for hydrophilic phycotoxins, while the extraction method for CIPS appeared more efficient for lipophilic compounds, but further investigations are required to investigate potential matrix effects. The utility of the novel CIPS was preliminarily demonstrated in the laboratory, with seemingly relatively improved performance for particular phycotoxin classes (Azaspiracids, Dinophysistoxins/Pectenotoxins, Microcystins, and Brevetoxin) as compared to SPATT. The expanded UPLC methods and CIPS must be trialed in the field next to determine their applicability to monitoring, research, and management. The benchtop work, however, already suggests that users should consider the full range of phycotoxins present at an environmental site and create sampling schemes appropriate for their region given the different sorption and extraction efficiencies of these two samplers.

## 1. Introduction

Phycotoxins are a group of bioactive compounds produced by numerous species and strains of harmful algae globally [1,2,3]. These chemicals can generally be classified into different classes based on their structures and health effects [4,5] and are incredibly multifarious in regard to their structure, polarity, distribution, stability, and concentration [5,6,7]. Phycotoxins result in decreasing motility [8,9] to mortality [10,11] from bivalves and fish, to birds and walruses [12,13,14]. Phycotoxins also lead to human illness through the consumption of contaminated seafood and similarly result in a range of adverse health effects from digestive issues to death [3,15,16,17]. Due to the threat phycotoxins pose, there is a high priority for the development, expansion, and use of novel monitoring and analytical approaches to protect public health. Harmful algal blooms (HABs) are a growing problem demonstrated by an increase in geographical and taxonomic diversity [15,18,19] and are expanding into seasons and areas where they previously did not occur, such as certain species shifting north in response to large-scale environmental changes [20,21]. In both marine and freshwater environments, co-occurrence of phycotoxin analogs or phycotoxin classes is commonly observed [22,23] and frequently occurs in estuarine areas where there is a confluence of fresh and marine water [24,25]. As blooms become more common and new threats from co-occurring phycotoxins become better understood, it is increasingly important to develop and expand upon existing monitoring and analytical methods to be able to handle the challenges presented.

In order to answer the questions posed by harmful algal blooms, multidisciplined approaches for sampling and phycotoxin quantification need to be undertaken. Water and animal tissue are two common types of samples used to investigate phycotoxin presence and concentration. However, due to the patchiness of blooms, discrete water sampling could miss phycotoxins, or the concentrations could be too low for detection. Determination of phycotoxins in edible meat is the gold standard for assessing threats to human health and tissue samples are routinely collected but cleanup steps and quantification methods are time-consuming and expensive. An alternative type of sampling is the use of passive samplers, which allows for continuous sorption of the analyte of interest onto a receiving phase deployed in the sampling media over a period of time. These samplers come in a range of sorbents, are typically deployed for 1–2 weeks in the water column, and are used extensively in multiple fields of study for a large range of water contaminants [26,27,28,29]. Passive samplers have demonstrated value for phycotoxins when used for long-term monitoring, detecting trends in presence or relative amounts over multiple seasons [30,31,32], or detecting emerging or low concentrations of phycotoxins in a region [33].

As with any tool, passive samplers have limitations to their utility, including having different affinities for phycotoxins based on sorbent type, and desorption based on ephemeral bloom and water concentrations, environmental conditions, and changing flow rates. Solid phase adsorption toxin trackers (SPATTs), for example, have been shown to have relatively low affinity for hydrophilic phycotoxins and some phycotoxins desorb within days [33,34,35], suggesting the sorbent is sensitive to rapidly changing environmental conditions. And without infused internal standards to account for this loss over time, SPATTs cannot be reliably used to determine absolute water concentrations of phycotoxins in the aquatic environment. The novel passive sampler, called the composite integrated passive sampler (CIPS), was therefore developed [36] with the aims of increasing reactivity with hydrophilic contaminants, and internal standards for determination of water concentrations [37].

The foci of this study were (1) to provide an expansion of a previously published UPLC-MS/MS method for the purpose of detecting and quantifying additional phycotoxins, and (2) to provide a comparison of phycotoxin sorption and extraction using two different passive samplers. Multiple new phycotoxin classes and congeners were included in the improved mass spectrometry method. Additionally, streamlined extraction processes are presented for two different passive samplers to allow for easy quantification of 24 freshwater and marine phycotoxins from passive sample extracts.

## 2. Results

### 2.1. Finalized Chromatographic Conditions

**Figure 1:**
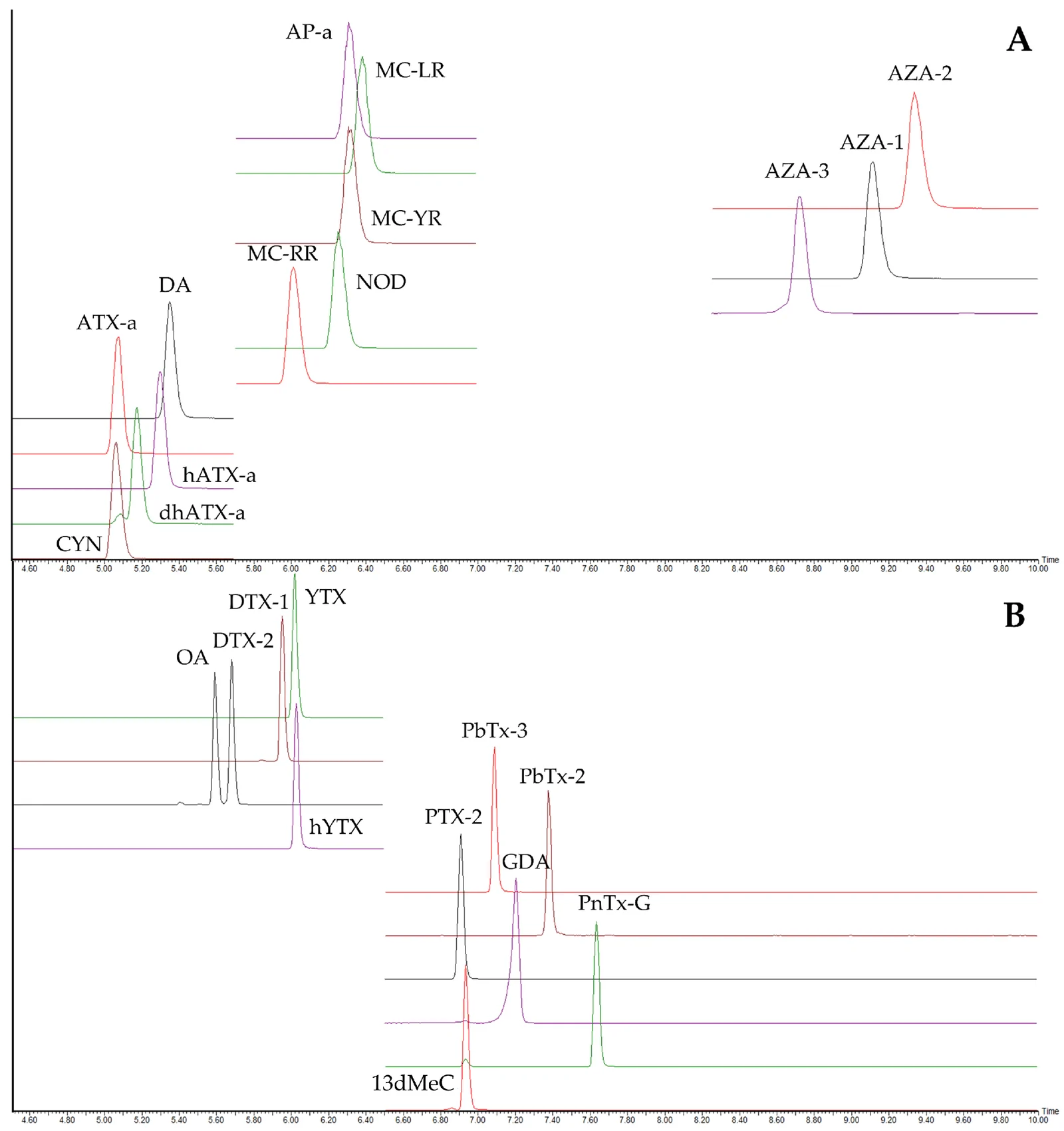
Chromatograms obtained from continuous acquisition of mass spectrometry data for all phycotoxins in a 100 µL injection of a mixed-phycotoxin standard on a common retention timescale using the two finalized methods under (**A**) acidic conditions and (**B**) basic conditions.

The UPLC-MS/MS methods described previously [38] were expanded to include more freshwater phycotoxins and recently commercialized marine phycotoxins by modifying the acidic and basic methods used. Anabaenopeptin-a (AP-a), anatoxin-a (ATX-a), homoanatoxin-a (hATX-a), dihydroanatoxin-a (dhATX-a), azaspiracid-3 (AZA3), cylindrospermopsin (CYN), and nodularin-R (NOD) were inserted into the acidic method while 13-desmethyl spirolide C (13dMeC), pinnatoxin-G (PnTx-G), brevetoxin-3 (PbTx3), and homoyessotoxin (hYTX) were inserted into the basic method. We maintained the ability to quantify all 24 phycotoxins within two injections using the trap/elute and at-column dilution (ACD) method for large volume injections (Figure 1). To achieve this, we tested modifications to loading time, trapping column, analytical column, mobile phase composition and ratios, and elution mobile phase flow rate.

For the acidic method, an Oasis HLB Direct Connect HP Column was determined to be the best trapping column, while using an Acquity UPLC HSS T3 Column along with a matching VanGuard pre-column as the analytical column was most suitable for all analytes in the acidic method. The temperature used for the acidic method was set to 40 °C as that was the maximum operating temperature for the column type. To quantify all the analytes included in the acidic method the sample loading time was set at 1 min and the elution flow rate was set at 0.15 mL/min. All compounds could not be detected with load times of 2 or 3 min and 0.15 mL/min gave the highest peak response with shortest gradient (Table A1). 50 mM (0.2%) FA + 2 mM ammonium formate in water (A) and acetonitrile (B) was used for mobile phase. The starting composition was 98% A and 2% B and changed from 2% B to 100% B over 5 min; it held at 100% B for 4 min to allow for elution of all analytes (Figure 1A).

Minimal changes were required to detect and quantify the phycotoxins added to the basic method. An amount of 6.7 mM (0.1%) ammonium hydroxide + 2 mM ammonium formate in water (A) and acetonitrile (B) was used for the mobile phase. The addition of buffer helped to stabilize the retention times. All phycotoxins in the basic method, including the four new analytes, were successfully separated and detected with the inclusion of the buffer (Figure 1B).

### 2.2. Method Performance Characteristics

Once separation and detection of the analytes was established, linear fit and percent relative standard deviation (%RSD) were checked to ensure method suitability for the purposes of this study. Test runs were conducted on both methods consisting of repeated injections of a mixed-phycotoxin standard at 0.5 µg/L (0.02 µg/L for AZAs) to evaluate repeatability and calculate LODs for all analytes. Then, a 9-point standard curve ranging from 0.1 µg/L to 50 µg/L (0.004 µg/L to 2 µg/L for AZAs) was run to ensure linearity. The highest LOD was 0.1 µg/L for microcystin-YR (MC-YR) with all other phycotoxins having LODs below the lowest concentration of the standard curve tested (Table A2). The standard curves were used to conduct linear regression analyses for all phycotoxins to determine the squared correlation coefficient (R^2^). All analytes were linear across the range tested apart from 13dMeC which was linear from 0.1 µg/L to 25 µg/L. Finally, short-term repeatability was measured using repeat injections from the start of the run, but was limited to testing only a single concentration. All %RSDs were <5% with the exception of brevetoxin-2 (PbTx2), microcystin-LR (MC-LR), and MC-YR with values of 5.56%, 5.90%, and 6.12%, respectively (Table A2). The results of these analyses indicate the UPLC-MS/MS approach is more efficient than single congener or single class methods and allows for the analysis of all 24 phycotoxins in the newly described method.

### 2.3. SPATT vs. CIPS Extraction Efficiency

A streamlined extraction procedure for SPATT was tested alongside an extraction procedure for CIPS to compare the ability to remove all sampler-sorbed phycotoxins. Phycotoxins were loaded onto the SPATT and CIPS in an artificial seawater solution for 24 h before conducting the extraction procedures. The SPATT was extracted with two 1.5 mL aliquots of 100% methanol (MeOH), intentionally following the common extraction method described in the literature [39,40,41,42]. The CIPS extraction method was modified to use 50:50 methanol:acetonitrile instead of published methods using dichloromethane:acetone [37,43] to allow for solvent compatibility with the polyvinylidene fluoride filters being used. The CIPS was extracted with one aliquot of 3 mL of a 50:50 mix of methanol:acetonitrile acidified to 0.5% with formic acid, in conjunction with a moisture-absorbing polymer to remove excess water.

**Figure 2:**
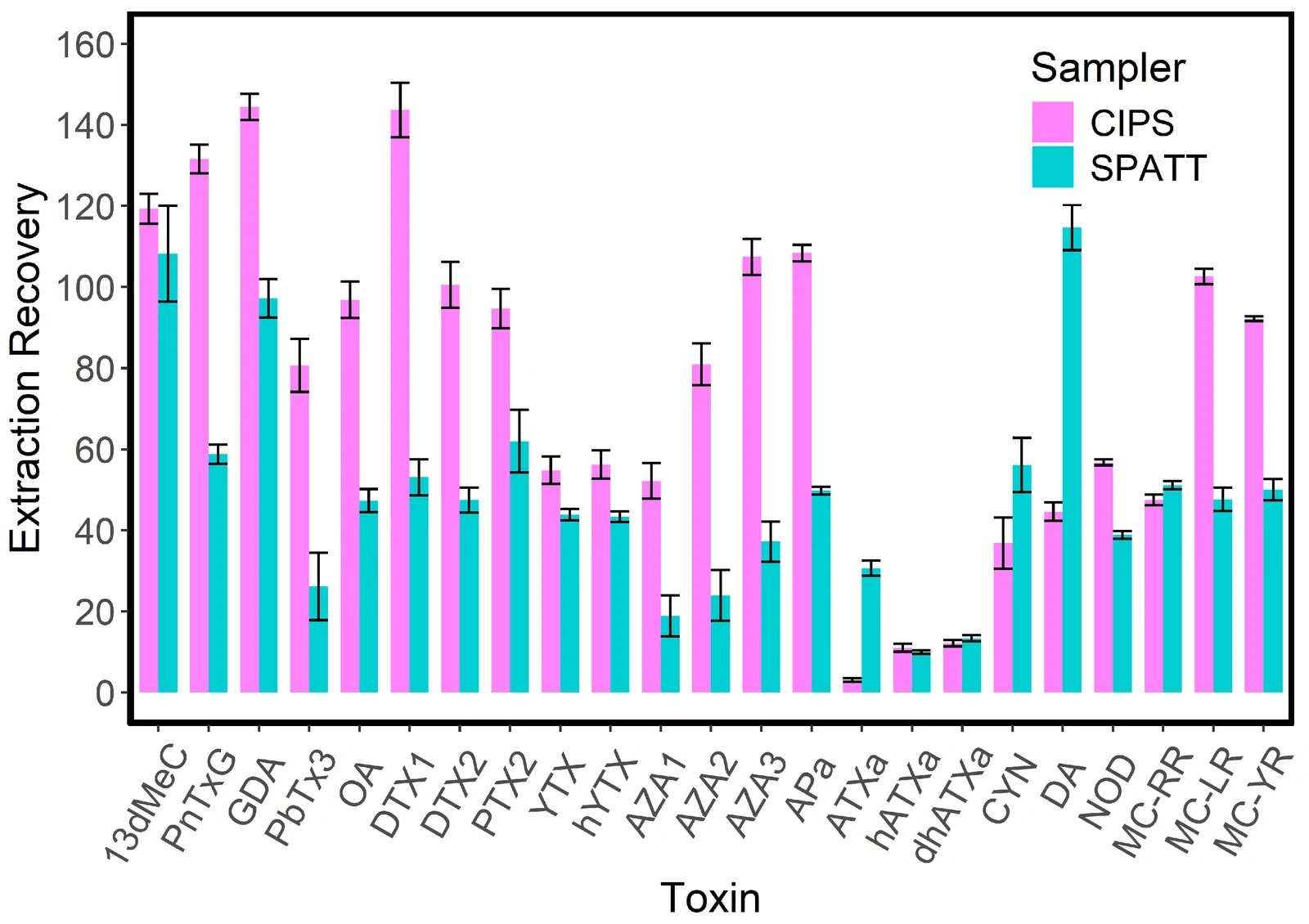
Percent of phycotoxins extracted from the two passive samplers, the CIPS and SPATTs, after organic solvent extraction (*n* = 3). Bars represent standard errors.

Quantification of all phycotoxins, in artificial seawater and extracted from the two samplers, was done using the newly described acidic and basic methods. Overall, more than 90% of each phycotoxin sorbed to the SPATT within 24 h, except for three hydrophilic phycotoxins, ATX-a, CYN, DA, and one lipophilic phycotoxin, PbTx3, which showed reduced sorption to this sampler (Table 1). The CIPS had relatively less phycotoxin sorbed after the 24 h, ranging from 21.9% to 90.6% sorbed from the seawater solution (Table 1). The CIPS tended to have a higher percentage of phycotoxin extracted from the sampler compared to SPATT (Figure 2). However, matrix effects during the quantification of the artificial seawater samples could result in enhanced extraction recoveries due to how it was calculated (Equations (3) and (4)). When calculating extraction recovery, potential degradation or loss to sampling materials was not included. However, reaction vials and transfer pipettes were rinsed with the extraction solvent to minimize loss and, based on previous research, we expected degradation to be minimal over the 24 h experimental period at room temperature in the dark [44,45,46,47,48,49,50].

## 3. Discussion

### 3.1. Detection Method Expansion

The modified UPLC-MS/MS method allowed for the quantification of 11 additional phycotoxins in the same extract, taking the total to 24 analytes, while still utilizing only two sample injections. Given that HABs are occurring more frequently and often concurrently, methods are needed to quantify phycotoxins from multiple HAB species across a large range of polarity. Paralytic shellfish toxins could not be included due to the chemistry of the group. These phycotoxins exhibit a wide range of stability and hydrophilicity and typically require pre- or post-column oxidation for quantification along with the use of specialized analytical columns [51,52]. The described methods herein can be used for targeted analysis when researchers have knowledge of the phycotoxins present in their environment, but can also be used as a screening tool to conduct a preliminary investigation when it is not known what phycotoxins are present or emerging in an aquatic environment.

For monitoring or research programs with limited knowledge of local phycotoxin presence, the combined passive sampler and detection methods described herein could potentially be utilized to conduct initial screenings and gain a baseline of possible threats to human and animal health. Even areas that have a long history of monitoring for phycotoxins could still be threatened by range expansion of new HAB species and their associated phycotoxins [20,21]. Detection and sampling methods that are able to quantify a large suite of freshwater and marine phycotoxins can help make these ambitions more streamlined and cost-effective.

### 3.2. Passive Sampler Comparison

The SPATT and CIPS are passive samplers able to sorb contaminants in situ throughout a deployment period, typically consisting of one to two weeks. However, their kinetic behaviors are likely different due to sorbent type and sampler construction. SPATT was constructed of a lipophilic mixture of styrene/divinylbenzene resin (Diaion^®^ HP20) held in place between pieces of 100 µm mesh [53]. The CIPS differs as it was utilized as a single sheet of adsorption media built to contain hydrophilic and lipophilic regions [36]. It is hypothesized that the lipophilic moieties in the HP20 resin (used in SPATT) are optimal for sorbing lipophilic phycotoxins such as okadaic acid (OA) and azaspiracids (AZAs), but do not strongly sorb hydrophilic phycotoxins, such as CYN and DA [54]. Alternatively, the hydrophilic/lipophilic structure of the CIPS material was hypothesized to allow for better binding across all polarities. The CIPS was evaluated herein for a range of phycotoxin contaminants and has been used to investigate a range of organic compounds [37,43].

Interestingly, during the benchtop study, SPATT sorbed more of each phycotoxin than the CIPS over the 24 h incubation in artificial seawater, with 0.5–21.4 ng more phycotoxin sorbed to SPATT. This was true for all phycotoxins tested except one compound, hydrophilic DA, for which more sorbed to the CIPS, 3.2 ng. The elevated sorption to SPATT resin could be due to the short incubation time used in this study (24 h) or potentially relatively slower sorption kinetics for the CIPS; as such, a longer laboratory or field application might improve sorption of all phycotoxins to the CIPS. Additionally, a study examining sorption kinetics of the two samplers is needed to fully elucidate differences before field application can be fully assessed. Another possible explanation for differences in sorption between the two samplers is that SPATT surface area can allow for high amounts of phycotoxin sorption per g of resin. This has been shown for one phycotoxin class, microcystins, at 18,400 ng/g [55]. This elevated saturation point suggests SPATT HP20 resin may easily sorb additional phycotoxins, especially in the laboratory when experimental solutions are relatively clean. Saturation points for the CIPS remain unknown. The SPATT resin has a surface area of 500 m^2^/g whereas the CIPS material has a surface area of 240 m^2^/g. This supports the notion that SPATTs can sorb more phycotoxin in shorter times. Future studies are required to investigate and compare how the various sampler structures affect the kinetics and saturation points of the two samplers, which will help to determine differences in rates and capacity during field deployments.

Passive samplers can be classified as equilibrium or kinetic samplers depending on if the receiving phase concentration equals the water concentration during the deployment period [26]. Despite not reaching equilibrium with the environment, kinetic samplers can be used to calculate time-weighted average concentrations, but require extensive laboratory calibration [56,57,58] since environmental factors have been shown to affect the rate of contaminant sorption onto passive samplers [59,60,61,62]. This has proven to be extremely difficult for SPATT due to great variability in phycotoxin physicochemical properties, environmental factors, and large ranges in deployment times [35,63]. Kinetic samplers start with a linear uptake phase before becoming more logarithmic [26,64]. Phycotoxins such as MCs, OA, PTX2, and YTX have shown linear uptake in deployments up to 8 days [53,65,66], indicating they are kinetic samplers. SPATTs can be constructed with multiple types of resin or as different deployment styles (e.g., disk vs. bag), and can be deployed for various lengths of time, making it difficult to use SPATTs to calculate environmental concentrations [35,63]. Phycotoxins also behave differently in the environment than legacy contaminants for which passive samplers were originally developed. Dissolved phycotoxin concentrations are influenced by multiple ephemeral sources such as release from cells during blooms or resuspension from sediment [67,68,69]. Additionally, some phycotoxins undergo relatively faster degradation rates than common, anthropogenic persistent organic pollutants, with half-lives ranging from only 2 days to 3 weeks [44,48,69,70], adding even more complexity to determining water concentration from SPATT.

**Figure 3:**
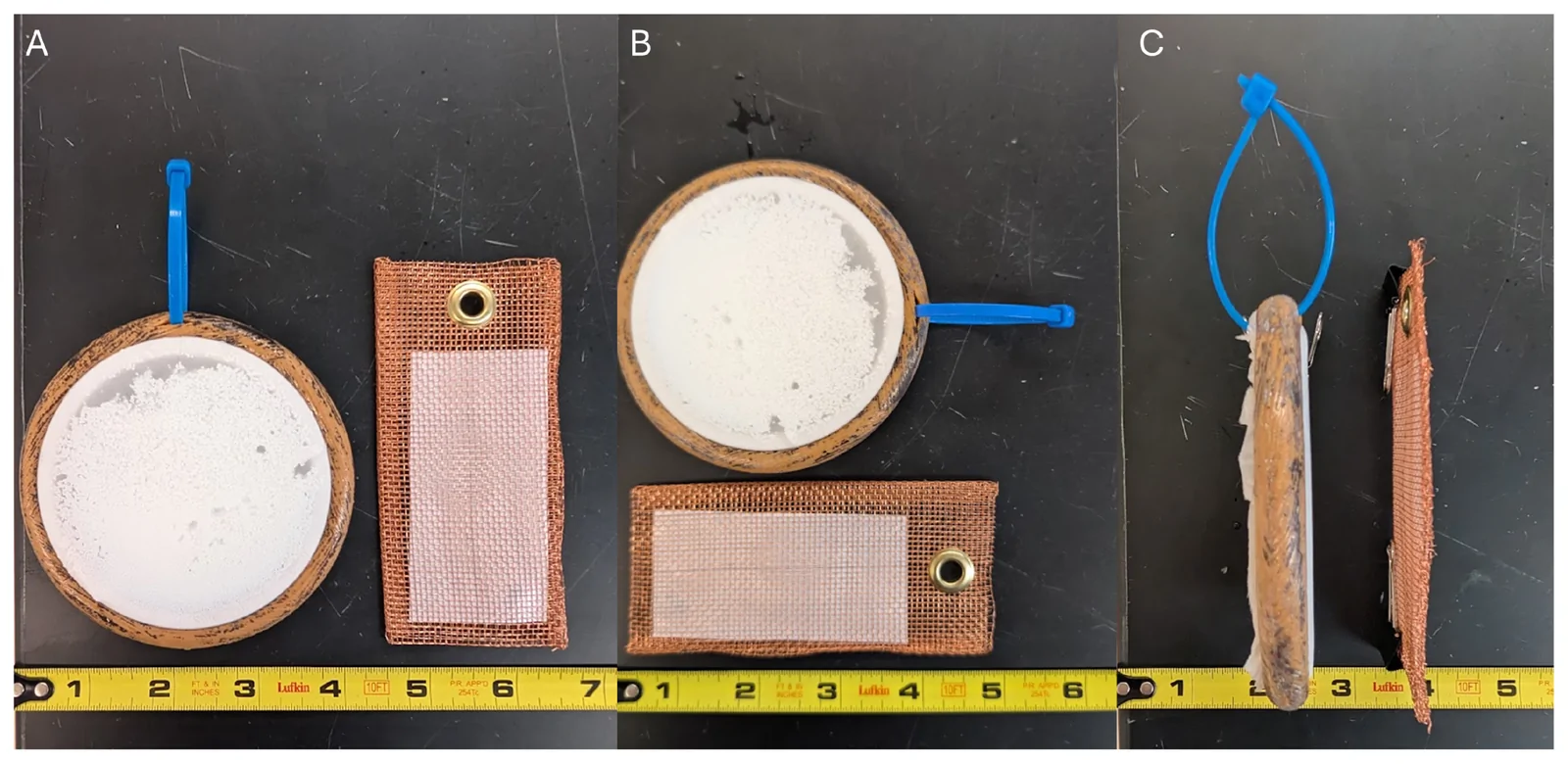
Pictures showing the (**A**) widths of the SPATT (**left**) and CIPS (**right**), (**B**) lengths of the SPATT (**top**) and CIPS (**bottom**) and (**C**) heights of the SPATT (**left**) and CIPS (**right**).

On the other hand, the CIPS, which appears to behave as a kinetic sampler, can be infused with performance reference compounds prior to field deployment. As the contaminant sorbs to the passive sampler, the performance reference compounds desorb, which can be used to calculate a time-weighted concentration of the contaminant in the water [26,64,71]. The environmental factors affecting the sorption of the contaminant also affect the desorption of the reference compounds and this provides additional information to allow for the calculation of the environmental concentration of the contaminant during the period when the sampler was deployed [64]. However, the desorption of the performance reference compounds must follow first-order kinetics in order to calibrate the sampler and accurately determine environmental concentrations [26,64,72]. This study has demonstrated that the CIPS sorbent binds a large range of phycotoxins and that sorbed phycotoxins can subsequently be extracted from the polymer in a single extraction and quantified. Future studies should investigate the behavior of the infused standards to evaluate the potential for the CIPS to be used for calculating environmental concentrations. Within 24 h, the CIPS sorbed more than 50% of the spiked phycotoxins for all compounds except CYN and DA which demonstrates the ability of the sampler to sorb a wide range of phycotoxins. Since the CIPS can be infused with performance reference compounds, it might be easier to perform laboratory calibrations and use them for calculating environmental concentrations of phycotoxins. The SPATT and CIPS (Figure 3) are constructed differently and phycotoxins sorb to the surface of the SPATT resin; whereas, the CIPS is mesoporous which suggest that phycotoxins can get embedded in the sampler. Investigations into how the porous structure of the CIPS affects desorption of phycotoxins should be done, which could add further support for their utility. Additionally, future studies should investigate the sorption characteristics of the CIPS in the laboratory and the field to determine its potential for implementation into monitoring and research programs.

Overall, the data demonstrated that the extraction method used with the CIPS resulted in higher extraction recoveries than the method for the SPATT (Table 1). It should be noted that matrix effects were observed when calculating phycotoxin concentrations in the artificial seawater samples. The combination of matrix effects and how extraction efficiencies were calculated could result in the enhancement of extraction efficiencies, particularly those with values over 100%. Despite this, it was still demonstrated that a wide range of phycotoxins are able to sorb to the material used in the CIPS and can subsequently be extracted for the purposes of detection and quantification. The three anatoxins involved in the study demonstrated relatively high sorption to both the SPATT and CIPS (>62.5%), but low extraction (<30.6%). These phycotoxins are hydrophilic and indicate that, even though both samplers can sorb the phycotoxins, the lipophilic extraction methods should be modified if future users plan to monitor this class of phycotoxins with either of these passive samplers. Additionally, since the same extraction methods were not used, direct comparisons are difficult to make. The CIPS utilizes a combined methanol:acetonitrile extraction mixture, which is more lipophilic than 100% methanol and could be hypothesized to result in higher extraction of lipophilic compounds such as OA. There is also the addition of the moisture-absorbing polymer, which should remove excess water, further increasing the lipophilicity. Overall, it was demonstrated that the SPATT and CIPS can sorb a wide range of phycotoxins that can be subsequently extracted with their respective procedures. Furthermore, these extraction methods could be modified to improve extraction efficiency of target phycotoxins in a region.

A single-step extraction was evaluated for each sampler to quantify all 24 phycotoxins within a single extract; successful quantification of all phycotoxins was achieved for both methods. Of interest is how different extraction efficiencies occurred between congeners within the same phycotoxin class. For example, AZA1, AZA2, and AZA3 for the CIPS method resulted in extraction recoveries of 52.2%, 81.0%, and 107.5%, respectively. Despite minor alterations in phycotoxin structure, polarities can vary, adding complexity into sampling, extraction, and quantification methods. The phycotoxins with the lowest extraction recoveries for the CIPS (ATX-a, hATX-a, dhATX-a, CYN, DA, and MC-RR) were also the most hydrophilic. Future studies might consider lowering the organic fraction of the extraction solvent (70% to 90%) for the CIPS or removing the moisture-absorbing polymer to determine if the extraction of hydrophilic phycotoxins can be increased without reducing the extraction of lipophilic compounds. Using a single-step extraction is ideal since it allows for quantification of all phycotoxins within one sample. Monitoring programs should consider their geographical region and which phycotoxins are of interest when choosing a sampler and extraction method. Overall, the CIPS, and its associated extraction method, is an efficient and effective option that can be used to determine which phycotoxins are present within a region if background data do not exist.

## 4. Conclusions

Previously published UPLC-MS/MS methods were improved to allow for detection and quantification of 11 additional phycotoxins, resulting in two methods that can quantify 24 phycotoxins across marine and freshwater environments. The methods were evaluated for a range of parameters and deemed suitable to detect all phycotoxins investigated in this study. The analytical methods were then applied to a laboratory experiment to compare two types of passive samplers for their ability to sorb and be extracted for the 24 phycotoxins. To demonstrate the potential high-throughput analysis, a single-step extraction method was evaluated for both passive samplers which would allow for quantification from a single extract. This study demonstrated that 24 phycotoxins could be sorbed to both passive samples and subsequently detected in the extract using the described methods. The CIPS was shown to sorb a wide range of phycotoxins that could then be extracted, providing an alternative passive sampling method to SPATT. Additionally, these methods are intended to be used for research or monitoring purposes and are not recommended for use in making regulatory decisions without proper and documented validation and certification.

## 5. Materials and Methods

### 5.1. Reagents and Analytical Phycotoxin Standards

All chromatography solvents used were LC-MS grade (Honeywell Burdick & Jackson, Muskegon, MI, USA). Ultrapure water used was produced by a Milli-Q system (Millipore, Billerica, MA, USA). Formic Acid Optima LC/MS (Thermo Fisher Scientific, Waltham, MA, USA), ammonium hydroxide eluent (≥25%, Honeywell Fluka, Charlotte, NC, USA), and ammonium formate Optima LC/MS (Thermo Fisher Scientific, Waltham, MA, USA) were used as additives for LC-MS mobile phases. Formic acid 98% (EMD Millipore, Merck, Darmstadt, Germany) was used in a wash solution.

The following phycotoxin certified reference materials were purchased from National Research Council Canada: 13-desmethylspirolide C (CRM-SPX1-b), anatoxin-a (CRM-ATX), homo-anatoxin-a (CRM-hATX), azaspiracid-1 (CRM-AZA1-b), azaspiracid-2 (CRM-AZA2-c), azaspiracid-3 (CRM-AZA3-b), cylindrospermopsin (CRM-CYN), dinophysistoxin-1 (CRM-DTX1-b), dinophysistoxin-2 (CRM-DTX2-c), domoic acid (CRM-DA-g), nodularin-R (CRM-NODR), okadaic acid (CRM-OA-d), pectenotoxin-2 (CRM-PTX2-b), pinnatoxin-G (CRM-PnTX-G), homo-yessotoxin (CRM-hYTX-b), and yessotoxin (CRM-YTX-c). Anabaenopeptin-A was purchased from Enzo Life Sciences (ALX-350-183-C100-100 µg). Dihydroanatoxin-A fumarate was purchased from Hello Bio (HB7494–1 mg). Brevetoxin-2 (PbTx-2, 203732-100 µg) and brevetoxin-3 (PbTx-3, 07576–0.5 mL) were purchased from EMD Millipore. A microcystin-RR, -YR, -LR mixed solution was purchased from Sigma Aldrich (33578-1 mL). Goniodomin-A was purified from *Alexandrium monilatum* by Drs. Constance and Tom Harris and provided by Dr. Kimberly Reece (VIMS).

### 5.2. Instrumentation

#### 5.2.1. Mass Spectrometry Conditions

A XEVO TQ-S Micro mass spectrometer (Waters, Milford, MA, USA) equipped with electrospray ionization (ESI) combined with multiple reaction monitoring (MRM) was used for detection. Capillary voltage was 3.00 kV, desolvation temperature was 500 °C, desolvation gas flow was 1000 L/h, collision gas flow was 0.15 mL/min, and source temperature was 150 °C. The mass spectrometer was operated in both ESI+ and ESI− modes.

Before adjusting the chromatographic conditions, direct infusions were performed for all phycotoxins (except hATX-a, dhATX-a, and PbTx-3) using a 500 µg/L concentrated solution. A 20 µg/L solution was used for the three azaspiracids. Waters Intellistart Software (MassLynx V4.2 SCN1049) was used when performing direct infusions to obtain four potential transitions along with cone voltages and collision energies for each phycotoxin with the top two being used for the MRM method. This software analyzes cone voltages from 2 to 100 V and collisions from 2 to 80 eV. The transitions which give the highest response over the tested range are highlighted by the software and voltages are optimized for the best four automatically. A combination of literature search and full scans were used to determine transitions for hATX-a, dhATX-a, and PbTx-3 [73,74] while collision and cone voltages were adjusted based on values from the ATX-a and PbTx-2 Intellistart results. Based on the results, the two precursor > product transitions with the highest response were chosen to be used in the MRM method, one for quantification, which was the dominant transition detected (Table 2), and one for confirmation (Table A3). For the three phycotoxins that did not undergo direct infusion, four transitions were chosen from the literature for each compound to be tested and the highest two were used in the MRM method.

#### 5.2.2. Chromatographic Conditions

A previously published UPLC method was expanded on to quantify additional phycotoxins [38]. The acidic mobile phases tested were ACN for organic (B) and ultrapure water for aqueous (A) with 50 mM (0.2%) FA + 2 mM ammonium formate as the additives tested. The basic mobile phases tested were ACN for organic (B) and ultrapure water for aqueous (A) with 6.7 mM (0.1%) ammonium hydroxide + 2 mM ammonium formate as the additives tested. The ammonium formate buffer was also added to the diluting and loading basic mobile phases (2 mM). Injection volumes of 100 µL were used for testing.

Besides the changes to the mobile phase, changes were also made to maintain a 16 min chromatographic run. To be able to detect the more hydrophilic phycotoxins (CYN and ATXs) added to the acidic method, multiple adjustments were required. First, the 3 min loading phase was reduced to 1 min, and an Oasis HLB Direct Connect trap was used instead of the BEH C18 Direct Connect. At 1 min, the valves switched. The linear gradient ran from 1 to 6 min, transitioning from 2% B to 100% B. From 6 to 10 min, mobile phase composition was held at 100% B before a linear gradient from 10 to 10.5 min changing from 100% B back down to 2% B. This was then held until the end of the chromatographic run (16 min).

### 5.3. Mass Spectrometry Development and Optimization

To improve analyte quantity, different combinations of trapping columns, analytical columns, loading times, and mobile phase flow rates and compositions were tested. All 24 phycotoxins were combined in either an acidic method or basic method mixed standard solution of 100% MeOH at a concentration of 10 µg/L. Confirmation of phycotoxin identification was verified through detection of two precursor > product transitions, reproducible retention times, and reproducible peak areas.

Initially, a literature review was conducted to determine if the new phycotoxins being added are typically quantified in LC-MS methods with acidic or basic mobile phase. AP-a, ATX-a, hATX-a, dihydroATX-a, AZA-3, CYN, and NOD were added to the previously published acidic method but could not be reproducibly quantified. To achieve detection for all acidic phycotoxins, an Oasis HLB Direct Connect HP Column, 2.1 × 30 mm, 20 µm, 80 Å was tested as a trapping column. The analytical column tested was an Acquity UPLC HSS T3 Column, 2.1 × 100 mm, 1.8 µm, 100 Å along with a matching VanGuard pre-column. The max temperature for this column is 40 °C; therefore the temperature used for the acidic method was reduced to 40 °C from 70 °C. All columns were purchased from Waters. Variations involving the loading phase time, elution mobile phase flow rate, and mobile phase starting composition were also investigated for the acidic method. Load times of 1, 2, and 3 min; elution flow rates between 0.15 and 0.5 mL/min; and starting compositions of 90%, 95% and 98% elution mobile phase A were tested in different combinations (Table A4) to determine which combination resulted in the highest sensitivity (Table A1).

For the basic method, the phycotoxins added to the basic method (13dMeC, PnTX-G, PbTx-3, and hYTX) fit within the previously established method. The addition of ammonium formate (2 mM) to the mobile phases was tested to see if retention time stability could be improved. The buffer (2 mM ammonium formate) was added to the loading, diluting, and eluting mobile phases.

All phycotoxins in the acidic method were evaluated using ESI+ mode. Addition of ammonium formate to the basic mobile phase shifted retention times slightly requiring ESI− to be used from 1 to 6.5 min and ESI+ from 6.5 to 10 min (Figure 1).

### 5.4. Method Performance Characteristics

After finalizing the chromatographic conditions, a test run was conducted for both acidic and basic methods to confirm short-term repeatability, determine linear fit, and establish instrument detection limits. The test run involved running a series of repeated injections using a mixed-phycotoxin standard at a concentration of 0.5 µg/L (0.02 µg/L for AZAs) followed by 9-point standard curves ranging from 0.1 µg/L to 50 µg/L for all phycotoxins except for the three azaspiracids, which were evaluated using a curve from 0.004 µg/L to 2 µg/L.

#### 5.4.1. Repeatability

To check the short-term repeatability, seven consecutive injections of a 0.5 µg/L (0.02 µg/L for AZAs) mixed-phycotoxin standard in 100% MeOH were run using acidic and basic methods. The peak areas for each analyte were used to quantify the standard deviation, which was then used to calculate the %RSD (Table A2). This was calculated using the following formula:

%RSD = 100 × standard deviation/average peak area(1)

#### 5.4.2. Limits of Detection

The instrument limits of detection (LODs) for each compound were determined mathematically by a series of repeated injections [75]. A mixed-phycotoxin standard solution of approximately 10× the estimated LOD was prepared in 100% MeOH. Seven repeated injections of this standard (0.5 µg/L or 0.02 µg/L for AZAs) were run, and peak areas were used to calculate the standard deviation. LOD peak area was calculated as 3.14 × standard deviation, and this was used to calculate the LOD concentration (Table A2) using the following equation:

LOD concentration = LOD peak area × sample concentration/average peak area(2)

#### 5.4.3. Linearity

Linearity was investigated by determining the linear fit over the range of standards tested by plotting the average peak area vs. concentration of the standard and was done in triplicate. Concentrations ranging from 0.1 µg/L to 50 µg/L were used for all phycotoxins except for the three azaspiracids, which were evaluated using a curve from 0.004 µg/L to 2 µg/L [76]. Regression analysis was conducted and standard curves were accepted as linear over the range evaluated if the squared correlation coefficient (R^2^) was >0.99.

### 5.5. Extraction Recovery of Passive Samplers

Two passive samplers were compared in this study to investigate their differences in phycotoxin sorption and extraction efficiency (Figure 3). The first samplers (SPATTs) have been used since 2004 [53]. The second passive sampler (CIPS) has been recently developed and is being tested to determine its suitability for potential application in the field of HABs and phycotoxins. SPATTs use a resin, typically Diaion^®^ HP-20, held between two pieces of mesh to bind phycotoxins. The CIPS is a sheet of cross-polymer containing both hydrophilic and lipophilic moieties to bind a range of contaminants.

Many studies exist for the application of SPATTs to monitor phycotoxins within marine and freshwater environments [35,77,78,79,80]. Similar to other studies, 100% MeOH was used as the extraction solvent to quantify phycotoxins [41,53,80,81,82]. The extraction method for the CIPS was modified from previous studies [37,43] to be compatible with the PVDF syringe filters and extractions were done with a 50:50 mixture of methanol:acetonitrile acidified to 0.5% formic acid.

To compare sorption and extraction recoveries, 4 mL amber vials were filled with 2 mL of buffered artificial seawater, at pH 8, salinity of 30, and room temperature. Phycotoxins were spiked into seawater at a concentration of 25 µg/L (1 µg/L for AZAs). PbTx-2 was not included in the experiment due to its apparent instability in water, and DA was spiked at a concentration of 75 µg/L. To begin the incubation, 0.25 g of HP-20 resin and a 1 cm × 4 cm sized piece of CIPS were added to separate 4 mL vials, conducted in triplicate. Vials were placed on a shaker table for 24 h in the dark, after which the sampler material was moved to a separate 4 mL amber vial for extraction. Additionally, 2 mL of MeOH were added to the vial containing the artificial seawater to quantify (1) the amount of unsorbed phycotoxin, i.e., % remaining in the artificial seawater after 24 h, and (2) amount of phycotoxin that sorbed to the sampler, using formula 3. The extraction recovery of each phycotoxin for each extraction method was determined using formula 4. PbTx-3 was added into the method after the other phycotoxins and run as a separate experiment using the same process to prepare the artificial seawater with the same batch of commercial sea salt mixture.

% Sorbed to Sampler = (1 − ng in water/ng spiked) × 100(3)

% Recovery = ng extracted from sampler/(ng spiked − ng in water) × 100(4)

SPATTs were extracted using two 1.5 mL aliquots of 100% MeOH with 30 min of bath sonication for each aliquot. The two aliquots were combined, and 1 mL was syringe filtered using a 0.22 µm PVDF filter into an autosampler vial for analysis. The CIPS was extracted using a single 3 mL aliquot of 50:50 methanol:acetonitrile acidified to 0.5% formic acid with 50 mg of Dionex MAP and bath sonication for 60 min. Exactly 1 mL of this extract was also syringe filtered before quantification. An aliquot of the seawater sample was taken and brought up to 95% MeOH without syringe filtering and quantified for unsorbed phycotoxin. PbTx-3 in seawater was brought up to 80% ACN before quantification. Phycotoxins were quantified using a standard curve from 0.1 µg/L to 10 µg/L (0.004 µg/L to 0.4 µg/L for AZAs) and 0.1 µg/L to 25 µg/L for DA. Standard curves were prepared in the same solvent as the sample being analyzed.

### 5.6. Phycotoxin Enhancement in CIPS Water

Enhancement of DA in the CIPS water samples was extremely apparent. To determine the degree of enhancement, the CIPS was soaked in artificial seawater for 24 h and then an aliquot of seawater was spiked with DA and left at room temperature for an additional 24 h. The sample was then brought up to 50% MeOH and left for 24 h before being brought up to 95% MeOH for quantification to keep consistent with the original quantification method. DA was enhanced by a factor of 1.3 in the CIPS water extract as compared to in MeOH. The DA in the water from the CIPS extraction recovery experiment was corrected for this enhancement and then used to calculate the extraction recovery. The DA recovery was the largest and why only DA enhancement was investigated without systematic investigation of the other phycotoxins. Other phycotoxin recoveries above 100% may also be exhibiting enhancement due to matrix effects, calibration differences between solvent and extract matrices, or uncertainty in the sorbed-fraction denominator which represents a limitation for comparison of extraction recoveries.

## Figures and Tables

**Table 1:** Mean percentages of each phycotoxin sorbed to sampler after 24 h of incubation in seawater and recovered from samplers using their respective extraction methods. The recovery experiment was completed in triplicate (±SE).

Phycotoxin	% Sorbed to Sampler After 24 h		% of Phycotoxin Extracted from Sampler	
	SPATT	CIPS	SPATT	CIPS
AP-a	97.9 (±0.9)	82.3 (±3.4)	49.8 (±1.0)	108.4 (±2.0)
ATX-a	70.2 (±3.5)	65.1 (±2.8)	30.6 (±1.9)	3.1 (±0.4)
hATX-a	98.6 (±0.8)	62.5 (±1.4)	10.0 (±0.4)	11.0 (±1.0)
dhATX-a	90.5 (±1.3)	88.7 (±1.2)	13.4 (±0.7)	12.1 (±0.8)
AZA-1	96.2 (±1.5)	69.9 (±3.4)	18.9 (±5.0)	52.2 (±4.4)
AZA-2	94.3 (±2.6)	58.3 (±4.1)	23.9 (±6.3)	81.0 (±5.2)
AZA-3	95.6 (±1.8)	54.7 (±5.1)	37.2 (±5.0)	107.5 (±4.4)
CYN	56.3 (±2.4)	40.9 (±7.5)	56.1 (±6.7)	36.9 (±6.3)
DA *	17.7 (±3.1)	21.9 (±6.1)	114.7 (±5.6)	44.6 (±2.3)
MC-RR	98.2 (±0.6)	85.1 (±2.7)	51.1 (±1.0)	47.5 (±1.3)
MC-LR	98.2 (±0.8)	86.3 (±2.5)	47.7 (±2.9)	102.6 (±1.9)
MC-YR	98.0 (±1.0)	83.3 (±3.6)	50.0 (±2.6)	92.2 (±0.6)
NOD	98.6 (±0.5)	90.6 (±2.0)	38.9 (±1.0)	56.8 (±0.8)
DTX1	99.1 (±0.5)	67.3 (±0.3)	53.1 (±4.4)	143.7 (±6.7)
DTX2	99.5 (±0.3)	83.3 (±1.3)	47.5 (±3.1)	100.6 (±5.6)
GDA	92.6 (±4.0)	49.8 (±5.1)	97.2 (±4.7)	144.4 (±3.2)
OA	99.5 (±0.3)	83.7 (±1.6)	47.4 (±2.8)	96.8 (±4.5)
PbTx3	69.9 (±25.1)	66.1 (±3.2)	26.2 (±8.3)	80.7 (±6.5)
PnTxG	94.7 (±2.4)	74.6 (±4.6)	58.8 (±2.3)	131.6 (±3.5)
PTX2	97.0 (±1.4)	63.5 (±4.5)	62.0 (±7.7)	94.7 (±4.8)
13dMeC	96.6 (±1.4)	77.0 (±4.0)	108.2 (±11.8)	119.3 (±3.7)
YTX	98.7 (±0.6)	62.1 (±4.9)	43.8 (±1.4)	54.8 (±3.4)
hYTX	98.6 (±0.8)	60.8 (±5.0)	43.4 (±1.4)	56.2 (±3.5)

* DA had enhancement in the CIPS water samples and % sorbed and % extracted values reflect correction described in Section 5.6.

**Table 2:** Finalized mass spectrometry parameters used across the two UPLC methods: basic and acidic.

Analyte	Ionization Mode	Adduct	Molecular Ion *m*/*z*	Cone Voltage (V)	Dominant Transition Detected	Collision Energy (eV)
AP-a	ESI+	H+	844.2659	30	844.2659 > 120.0549	78
ATX-a	ESI+	H+	166.0404	30	166.0404 > 149.0118	10
hATX-a	ESI+	H+	180.0400	30	180.0400 > 163.0600	14
dhATX-a	ESI+	H+	168.0900	30	168.0900 > 43.0100	26
AZA-1	ESI+	H+	842.3659	30	842.3659 > 824.4298	30
AZA-2	ESI+	H+	856.4298	30	856.4298 > 838.4884	30
AZA-3	ESI+	H+	828.3936	30	828.3936 > 810.3939	30
CYN	ESI+	H+	416.1428	30	416.1428 > 194.1149	34
DA	ESI+	H+	312.1066	30	312.1066 > 266.43	15
MC-RR	ESI+	2H+	519.9404	30	519.9404 > 135.0945	35
MC-LR	ESI+	H+	995.4559	30	995.4559 > 135.0945	85
MC-YR	ESI+	H+	1045.4659	30	1045.4659 > 135.0945	85
NOD	ESI+	H+	825.2898	30	825.2898 > 135.0891	66
DTX1	ESI−	−H	817.4574	20	817.4574 > 113.0330	64
DTX2	ESI−	−H	803.5213	20	803.5213 > 113.0316	60
GDA	ESI+	NH_4_+	786.4213	20	786.4213 > 139.1234	24
OA	ESI−	−H	803.5213	20	803.5213 > 113.0316	60
PbTx2	ESI+	H+	895.7289	20	895.7289 > 877.5100	10
PbTx3	ESI+	H+	897.5000	20	897.5000 > 725.4400	20
PnTxG	ESI+	H+	694.3936	20	694.3936 > 164.1742	58
PTX2	ESI+	NH_4_+	876.5213	20	876.5213 > 841.4846	16
13dMeC	ESI+	H+	692.3574	20	692.3574 > 164.1077	50
YTX	ESI−	2H−	570.6362	20	570.6362 > 467.4713	26
hYTX	ESI−	2H−	577.2851	20	577.2851 > 474.1792	24

**Figure A1:**
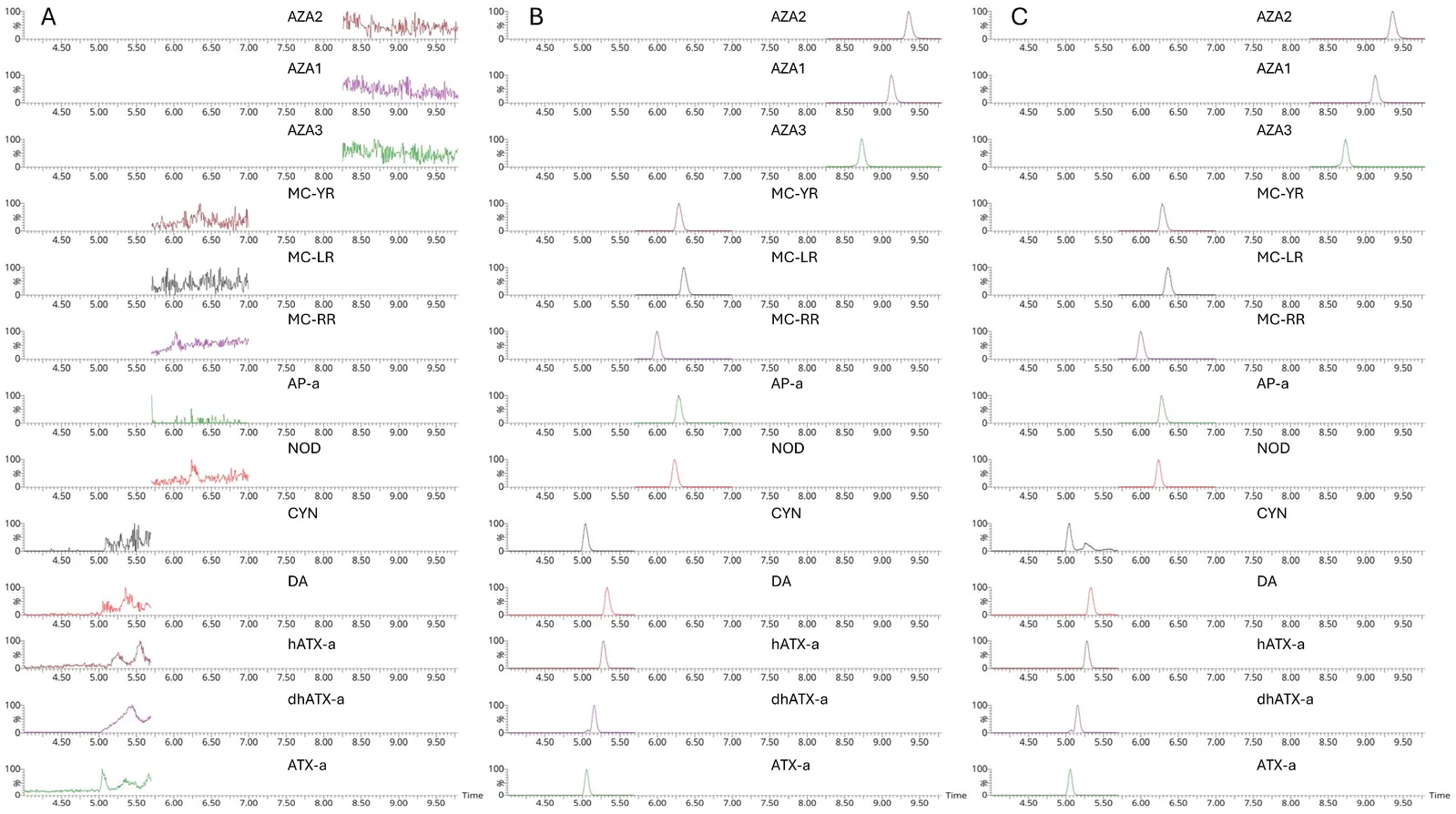
Stacked chromatograms obtained from continuous acquisition of mass spectrometry data for all phycotoxins in the acidic method in a 100 µL injection of a (**A**) methanol blank, (**B**) 10 µg/L mixed phycotoxin standard, and (**C**) representative extracted sample.

**Figure A2:**
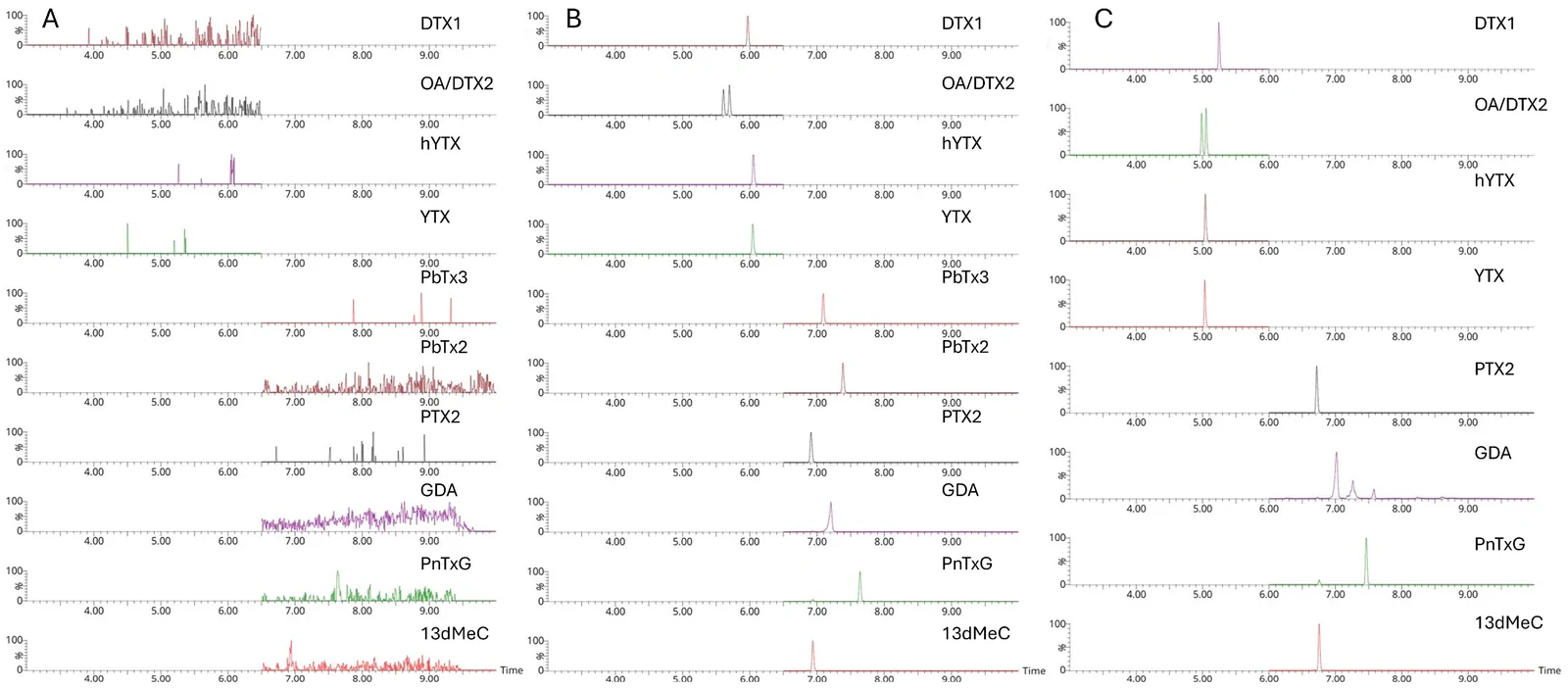
Stacked chromatograms obtained from continuous acquisition of mass spectrometry data for all phycotoxins in the basic method in a 100 µL injection of a (**A**) methanol blank, (**B**) 10/0.4 µg/L mixed phycotoxin standard, and (**C**) representative extracted sample. PbTx2 and PbTx3 are not included in (**C**) due to PbTx2 not being included in the extraction recovery experiment and PbTx3 being run separately.

## Data Availability

The data presented in this study are openly available in NOAA NCEI at https://www.ncei.noaa.gov/, reference number NA25NOSX478C0027.

## Abbreviations

The following abbreviations are used in this manuscript:

13dMeC	13-desmethylspirolideC
AP-a	Anabaenopeptin-a
ATX-a	Anatoxin-a
hATX-a	Homoanatoxin-a
dhATX-a	Dihydroanatoxin-a
AZA-1	Azaspiracid-1
AZA-2	Azaspiracid-2
AZA-3	Azaspiracid-3
CIPS	Composite Integrated Passive Sampler
CYN	Cylindrospermopsin
DA	Domoic acid
DTX-1	Dinophysistoxin-1
DTX-2	Dinophysistoxin-2
GDA	Goniodomin-A
LOD	Limit of detection
MC-LR	Microcystin-LR
MC-RR	Microcystin-RR
MC-YR	Microcystin-YR
NOD	Nodularin-R
OA	Okadaic acid
PbTx-2	Brevetoxin-2
PbTx-3	Brevetoxin-3
PnTx-G	Pinnatoxin-G
PTX-2	Pectenotoxin-2
PVDF	Polyvinylidene fluoride
SPATT	Solid Phase Adsorption Toxin Tracker
UPLC-MS/MS	Ultra-performance liquid chromatography-tandem mass spectrometry
YTX	Yessotoxin
hYTX	Homoyessotoxin

## Appendix A

**Table A1:** Phycotoxin peak area for the quantification transition of all analytes in the acidic method for the purpose of optimizing the chromatographic method.

Test Method	AP-a	ATX-a	hATX-a	dhATX-a	AZA1	AZA2	AZA3	CYN	DA	MC-RR	MC-LR	MC-YR	NOD
A	4368	8459	88,461	21,169	18,072	13,316	14,863	1937	12,552	25,624	15,511	12,052	69,251
B	1689	5090	50,619	12,919	10,930	7959	8979	774	5372	11,185	7811	6574	30,157
C	3955	8461	79,295	17,957	17,362	11,826	13,512	1476	10,270	21,715	14,231	10,965	64,223
D	3966	9828	88,604	22,359	18,328	12,856	12,967	1961	11,393	23,293	15,495	12,155	66,319
E	5810	11,349	96,738	23,213	24,620	16,752	16,230	2056	15,990	32,856	23,258	16,182	88,981
F	5278	7351	75,946	17,187	24,382	17,498	16,478	2045	14,849	31,634	20,873	15,136	81,445
G	3945	7627	62,201	14,131	20,844	15,331	17,045	942	10,924	24,626	15,467	12,721	55,712
H	9029	15,047	117,397	24,455	43,422	24,241	25,034	4107	27,311	54,135	32,090	22,058	110,890
I	7748	11,431	118,095	25,599	34,872	23,793	32,138	3666	27,082	52,926	24,283	19,108	102,369

**Table A2:** Calculated LOD and %RSD for each phycotoxin using the updated UPLC-MS/MS conditions.

Phycotoxins	LOD	%RSD
AP-a	0.03	2.19
ATX-a	0.02	1.18
hATX-a	0.03	1.66
dhATX-a	0.04	2.25
AZA-1	0.002	2.51
AZA-2	0.001	2.36
AZA-3	0.002	2.55
CYN	0.06	3.53
DA	0.06	3.53
MC-RR	0.03	2.13
MC-LR	0.09	5.90
MC-YR	0.10	6.12
NOD	0.03	1.83
DTX1	0.05	3.17
DTX2	0.03	1.99
GDA	0.04	2.41
OA	0.06	3.83
PbTx2	0.09	5.56
PbTx3	n/a	n/a
PnTxG	0.02	1.52
PTX2	0.07	4.28
13dMeC	0.03	1.82
YTX	0.06	3.73
hYTX	0.04	2.54

**Table A3:** Optimized mass spectrometer quantification and confirmation transitions for all phycotoxins in the basic and acidic method.

Analyte	Molecular Ion *m*/*z*	Cone Voltage (V)	Quantification Transition	Collision Energy (eV)	Confirmation Transition	Collision Energy (eV)
AP-a	844.2659	30	120.0549	78	637.2147	26
ATX-a	166.0404	30	149.0118	10	131.0401	16
hATX-a	180.0400	30	163.0600	14	145.0500	16
dhATX-a	168.0900	30	43.0100	26	93.0600	20
AZA-1	842.3659	30	824.4298	30	806.4253	40
AZA-2	856.4298	30	838.4884	30	820.4865	40
AZA-3	828.3936	30	810.3939	30	792.5000	40
CYN	416.1428	30	194.1149	34	336.1023	20
DA	312.1066	30	266.43	15	161.3300	20
MC-RR	519.9404	30	135.0945	35	70.0000	45
MC-LR	995.4559	30	135.0945	85	107.0400	70
MC-YR	1045.4659	30	135.0945	85	70.0000	90
NOD	825.2898	30	135.0891	66	104.7000	90
DTX1	817.4574	20	113.0330	64	255.1153	44
DTX2	803.5213	20	113.0316	60	255.1725	46
GDA	786.4213	20	139.1234	24	95.0657	50
OA	803.5213	20	113.0316	60	255.1725	46
PbTx2	895.7289	20	877.5100	10	109.0654	74
PbTx3	897.5000	20	725.4400	20	129.2700	70
PnTxG	694.3936	20	164.1742	58	676.2961	34
PTX2	876.5213	20	841.4846	16	823.4826	16
13dMeC	692.3574	20	164.1077	50	674.3229	30
YTX	570.6362	20	467.4713	26	396.3487	24
hYTX	577.2851	20	474.1792	24	403.4000	30

**Table A4:** Different combinations of flow rate, gradient composition, and gradient run time during acidic method chromatographic optimization.

Test Method	Load Time	Flow Rate	Starting Composition	Ending Composition	Gradient Run Time
A	1 min.	0.3 mL/min.	90% A 10% B	0% A 100% B	1–8 min.
B	1 min.	0.5 mL/min.	90% A 10% B	0% A 100% B	1–8 min.
C	1 min.	0.3 mL/min.	98% A 2% B	0% A 100% B	1–8 min.
D	1 min.	0.3 mL/min.	98% A 2% B	0% A 100% B	2–8 min.
E	1 min.	0.25 mL/min.	98% A 2% B	0% A 100% B	3–8 min.
F	1 min.	0.25 mL/min.	95% A 5% B	0% A 100% B	3–8 min.
G	1 min.	0.3 mL/min.	98% A 2% B	0% A 100% B	3–8 min.
H	1 min.	0.15 mL/min.	98% A 2% B	0% A 100% B	1–8 min.
I	1 min.	0.15 mL/min.	98% A 2% B	0% A 100% B	1–6 min.
